# Improving Diversity in a Rural Academic Obstetrics and Gynecology Training Program

**DOI:** 10.1097/og9.0000000000000018

**Published:** 2024-07-02

**Authors:** Christina DeAngelis, Sofia Espinosa, Tonya Wright

**Affiliations:** Penn State Milton Hershey Medical Center, Hershey, Pennsylvania.

## Abstract

Both the Accreditation Council for Graduate Medical Education and the American College of Obstetricians and Gynecologists advocate for greater diversity within our workforce, which starts with recruiting and retaining a diverse residency pool. Historically, applicants who are underrepresented in medicine have been more likely to apply to residency programs located in urban locations, as opposed to a rural training environment. In this commentary, we discuss a novel strategy used to attract underrepresented in medicine applicants to a rural academic obstetrics and gynecology training program. The strategy encompassed a virtual weeklong elective with the goals of bolstering their residency applications and showcasing our faculty and residency training.

## Abstract 2

A virtual elective for underrepresented minority medical students can improve diversity in a rural academic obstetrics and gynecology training program.

Although it is well established that patients benefit from a more diverse health care workforce, it is especially critical in the field of obstetrics and gynecology, where health care disparities are contributing factors to increased maternal morbidity and mortality.^1^ In 2020, the American College of Obstetricians and Gynecologists released a statement calling out racism as a public health crisis and voiced a commitment to take collective action to improve health equity.^2^ The Accreditation Council for Graduate Medical Education has undertaken common program requirements that hold residency programs accountable for the implementation of policies and procedures aimed at recruiting a diverse resident complement.^3^ Improved diversity in residency training is an important area to add to overall diversity in the physician workforce. Unfortunately, prior efforts to increase underrepresented minority candidates for obstetrics and gynecology residency training in our rurally located academic program have not been very successful, with only 6.25% of graduates over the past 10 years being underrepresented minority individuals.

## THE RATIONALE FOR RECRUITING DIVERSE APPLICANTS

The case to diversify the workforce in medicine is critical to the field of obstetrics and gynecology as we face a maternal health crisis with alarming racial differences in outcomes. In 2021, the maternal mortality rate for non-Hispanic Black women was 69.9 deaths per 100,000 live births, 2.6 times the rate for non-Hispanic White women (26.6).^4^ There are several well documented reasons why diversifying the workforce is of paramount importance, including advancing cultural competency of health care professionals who care for an increasingly diverse U.S. population, increasing access to high-quality health care services in underserved populations, and strengthening the medical research agenda for minority patients plagued by a history of distrust in the profession.^5,6^ Despite this, Black and Hispanic physicians, for example, represent 5.0% and 5.8% of all active physicians in the United States, even though these groups make up 13% and 19% of the population, respectively.^7^ Furthermore, there has been a decline in Black obstetrics and gynecology residents (10.2%–7.9%) in U.S. training programs and a stagnant rate of Hispanic (9.6%–10.1%) and Native American or Alaskan Native residents (0.2%–0.1%) over the period from 2014 to 2019.^8^ To better care for our diverse patients and decrease health disparities, we must recognize the obligation to increase the number of underrepresented minority physicians.^7^

## STRATEGY TO IMPROVE RECRUITMENT OF DIVERSE APPLICANTS

Our institution is committed to improving diversity and inclusion through recruiting faculty and trainees of diverse ethnic and racial backgrounds. Despite our traditional strategies, our efforts have been less than successful, and our greatest barriers continue to be our geographic location in a rural environment and distance to urban centers. The residency program has historically attracted highly competitive applicants of racially or ethnically nondiverse backgrounds who, on completion of residency training, pursue competitive fellowships, work in academic or community centers or join private practices.

We hypothesized that, if we offered a virtual elective for underrepresented minority third-year medical students interested in pursuing obstetrics and gynecology residency training, we may interest applicants who would not have otherwise considered our program based on its location. The focus of the elective was not to increase a participant's clinical knowledge but rather to provide mentorship to make their application to residency stronger and to share the training experience we offer at our institution. By offering this elective as a virtual experience, we eliminated any financial or logistical barriers, as there would be no cost for the applicant or our institution and there would be no disruption for the student during acting internships.

We used social media platforms (Instagram and Facebook) as well as direct outreach to the Latino Medical Student Association, the Student National Medical Association, and the Association of American Indian Physicians to advertise the virtual elective on their websites. Additionally, faculty at our institution reached out to contacts at various medical schools and sent information on the elective. We received 22 applications and selected eight individuals to participate using a standardized selection process. Those individuals who were interested submitted a short application providing some basic education and demographic information, as well as a response as to why they were applying. The applicants who had already completed their medical training through a non-U.S. medical school and were no longer medical students were not considered, which reduced the pool to eight individuals who were all invited to participate.

## THE VIRTUAL ELECTIVE

The elective spanned 1 week (four consecutive weeknights) during August of 2022, with 2-hour sessions each night. Sessions were led by a number of faculty across various divisions and covered a variety of topics (Table 1). The session goals were two-pronged: 1) provide the applicants with advice and mentorship through the Electronic Residency Application System process and, thereby, bolster their applications to all programs; and 2) convey site-specific information that could enhance their interest in applying to our program.

**Table 1.** Virtual Elective Agenda

Date	Topic	Presenter
Day 1	ERAS overview and application advice	Vice Chair of Education
Day 2	MFM, urogynecology, and MIGS specialty overview and Fellowship advice	Division Chiefs of MFM and urogynecology
Day 3	“The Inside Scoop” from the residents	Resident representatives PGY-1–PGY-4
Day 4	REI, gynecologic oncology, complex family planning specialty overview, and Fellowship advice and wrap-up	Division Chiefs of REI, gynecologic oncology, and complex family planning

ERAS, Electronic Residency Application System; MFM, maternal–fetal medicine; MIGS, minimally invasive gynecologic surgery; REI, reproductive endocrinology and infertility.

The Electronic Residency Application System mentorship sessions covered how to write strong personal statements, tips for virtual interviews to help applicants stand out, and counseling on how to use signals and make a rank list. Some sessions featured subspecialists who provided advice on what to look for in a program and how applicants could make themselves competitive for future fellowship training.

There was a resident-led session with the goal of providing the applicants with insight into the lives of trainees in our program, our work environment and culture, and navigating the trainee environment in our geographic location. Participants in the elective were not graded or subjected to any faculty or resident evaluation, nor did their participation factor into being offered an interview for residency training or their standing in the rank list if they interviewed.

## OUR SUCCESS STORY

For the 2023 National Board of Medical Examiners match cycle, we received four signals (three gold and one silver) from the eight underrepresented minority participants. “Signaling” in the obstetrics and gynecology match process allows applicants an equitable approach to demonstrate a sincere interest in specific programs.^9^ Applicants who participate in signaling can assign up to three gold signals and 15 silver signals to residency programs of their choosing.^9^ Gold indicates highest interest in a program and silver indicates very high interest in the program.^9^

**Fig. 1.**
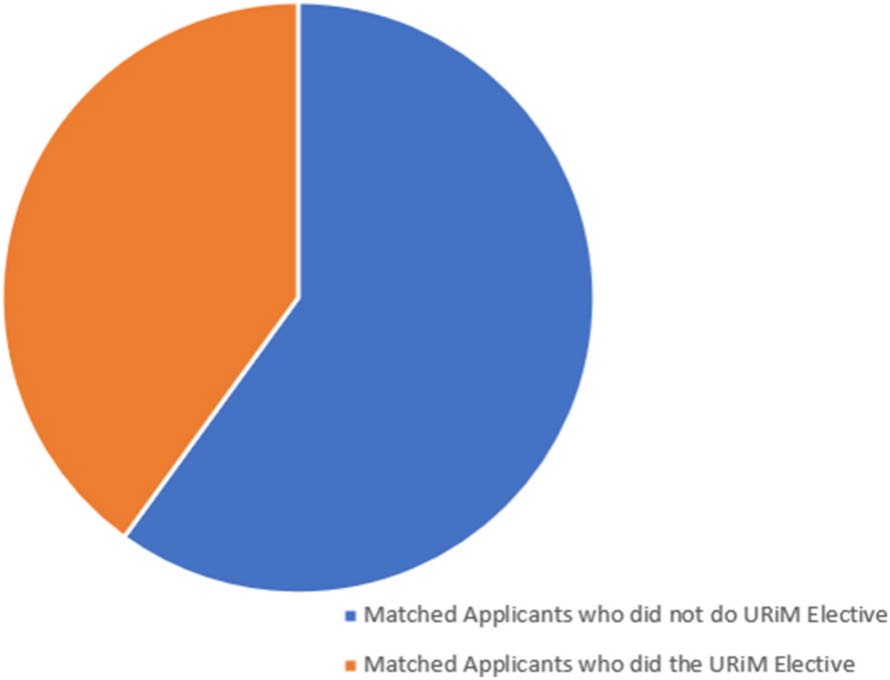
PGY-1 residency class, 2023. URiM, underrepresented in medicine.

Two of the four students who provided us with tokens successfully matched at our institution. The two matched applicants self-described as belonging to a racial or ethnic group that was underrepresented in medicine that we had not matched in the prior decade in our residency program. This resulted in a 40% rate of underrepresented minority residents in that incoming residency class (Fig. 1), in contrast to the prior 5 years, which only had one underrepresented minority resident out of a total complement of 20.

*DeAngelis. Improving Diversity in a Training Program. O&G Open 2024*.

All eight participants were anonymously surveyed after completing the virtual elective. All individuals responded favorably and reported that the elective was very good or excellent, and all stated that they would recommend the experience to a colleague. Survey responses indicated that all participants felt the elective was useful to bolster their application to be more competitive, allowed them to better assess whether a program would meet their professional goals, and increased their understanding of the training they would receive at our institution.

## CONCLUSION

Having a commitment to enhancing diversity at an institution that lacks it isn't achievable without trying novel approaches to make it a reality. Increasingly, the value of a diverse physician workforce, particularly in the field of obstetrics and gynecology, has been viewed as vital in improving the health care inequities in reproductive and maternal health. The use of social media to reach our target audience as well as the virtual nature of the elective also provided an equitable approach to our recruitment efforts—eliminating concerns for financial limitations, access, and nepotism.

Our commitment to recruitment through this novel approach and ultimately our success in matching two outstanding underrepresented minority students in our residency program sends a clear message of our support and dedication to eliminating disparities. Additionally, the faculty and residents who participated in the elective all found the experience to be highly rewarding, not just in enhancing our program success, but providing historically underrepresented students in medicine an opportunity to bolster their applications, thereby ensuring a successful match in the field as a whole.

However, without continued encouragement, residents, especially those who face additional challenges, may not thrive and reach their full potential without ongoing support. We are fortunate at our institution to have an organization jointly run by the Graduate Medical Education office and our academic institution called the Network of Under-represented Residents and Fellows. This group promotes cultural diversity through community involvement, mentorship with diverse faculty, professional networking as well as support for the recruitment of diverse medical students into residency programs. The underrepresented minority residents who have matched at our institution have used the Network of Under-represented Residents and Fellows as an adjunct to their support system and have engaged in mentorship within our department with our faculty who are best able to support them both professionally and personally.

We plan to continue to offer this underrepresented minority virtual elective and will seek out other innovative methods to create a more diverse workforce at our institution. We recognize that one year of experience provides for a limited ability to assess long-term outcomes, but we were encouraged by these initial results. We will continue to track outcomes with the virtual underrepresented minority elective and strive for additional opportunities to increase our diversity. If there is more emphasis placed by obstetrics and gynecology residency programs on offering experiences that are appealing and valuable for underrepresented minority students, there may be an increase in obstetrics and gynecology residency applications from this group. Sharing ideas and experiences in the graduate medical education community will make all of our programs stronger.
